# Development of a Food-Based Diet Quality Score from a Short FFQ and Associations with Obesity Measures, Eating Styles and Nutrient Intakes in Finnish Twins

**DOI:** 10.3390/nu11112561

**Published:** 2019-10-23

**Authors:** Guiomar Masip, Anna Keski-Rahkonen, Kirsi H. Pietiläinen, Urho M. Kujala, Mirva Rottensteiner, Karoliina Väisänen, Jaakko Kaprio, Leonie H. Bogl

**Affiliations:** 1 Department of Public Health, University of Helsinki, PO Box 20, Tukholmankatu 8B, 00014 Helsinki, Finland; guiomar.masip-manuel@helsinki.fi (G.M.); anna.keski-rahkonen@helsinki.fi (A.K.-R.); jaakko.kaprio@helsinki.fi (J.K.); 2 Obesity Research Unit, Research Program for Clinical and Molecular Metabolism, Faculty of Medicine, University of Helsinki, PO Box 63, Haartmaninkatu 8, 00290 Helsinki, Finland; kirsi.pietilainen@helsinki.fi; 3 Obesity Centre, Abdominal Centre, Endocrinology, Helsinki University Central Hospital and University of Helsinki, PO Box 340, Haartmaninkatu 4, 00290 Helsinki, Finland; 4 Faculty of Sport and Health Science, University of Jyväskylä, PO Box 35, Seminaarinkatu 15, 40014 Jyväskylä, Finland; urho.m.kujala@jyu.fi (U.M.K.); mirva.rottensteiner@ksshp.fi (M.R.); 5 Department of Medicine, Central Finland Health Care District, Keskussairaalantie 19, 40620 Jyväskylä, Finland; 6 School of Business and Services Management, JAMK University of Applied Sciences, PO Box 207, Rajakatu 35, 40101 Jyväskylä, Finland; karoliina.vaisanen@jamk.fi; 7 Institute for Molecular Medicine Finland (FIMM), University of Helsinki, PO Box 20, Tukholmankatu 8, 00014 Helsinki, Finland; 8 Leibniz Institute for Prevention Research and Epidemiology—BIPS, Achterstraße 30, 28359 Bremen, Germany; 9 Department of Epidemiology, Center for Public Health, Medical University of Vienna, Kinderspitalgasse 15, 1090 Vienna, Austria

**Keywords:** obesity, eating behaviors, diet quality score, nutrient intake, twins, short FFQ, BMI, waist

## Abstract

We constructed a food-based diet quality score (DQS) and examined its association with obesity measures, eating styles and nutrient intakes. Participants were 3592 individuals (764 dizygotic [DZ] and 430 monozygotic [MZ] twin pairs) from the FinnTwin16 study. The DQS (0–12 points) was constructed from a short 14 item food frequency questionnaire. Anthropometric measures and eating styles were self-reported. Nutrient intakes were calculated from food diaries completed in a subsample of 249 individuals (45 same-sex DZ and 60 MZ twin pairs). Twins were analyzed both as individuals and as twin pairs. The DQS was inversely associated with body mass index (β = −0.12, per one-unit increase in DQS, *p* < 0.001), waist circumference (β = −0.34, *p* < 0.001), obesity (odds ratio [OR]: 0.95, *p* = 0.004) and abdominal obesity (OR: 0.88, *p* < 0.001), independent of sex, age, physical activity and education. A higher DQS was associated with health-conscious eating, having breakfast, less snacking, fewer evening meals, and a higher frequency and regularity of eating. The DQS was positively correlated with the intakes of protein, fiber and magnesium and negatively correlated with the intakes of total fat, saturated fat and sucrose. Within twin pairs, most of the associations between the DQS with eating styles and some nutrients remained, but the DQS was not associated with obesity measures within twin pairs. The DQS is an easy-to-use tool for ranking adults according to diet quality and shows an association with obesity measures, eating styles and key nutrients in the expected direction.

## 1. Introduction

Because dietary patterns have a major role in the development of chronic diseases [1], accurate dietary assessment is important. Dietary assessment methods provide data on dietary intake and diet quality. Due to their feasibility and low cost in large-scale studies, food frequency questionnaires (FFQ) are frequently employed in large nutritional epidemiological studies [2,3]. Recent studies have shown that higher diet quality scores derived from extensive FFQs, such as the Alternate Mediterranean Diet, the Alternate Healthy Eating Index 2010 (AHEI-2010) and the Dietary Approaches to Stop Hypertension adherence scores, are associated with less weight gain over time [4]. Dietary intake is closely linked with eating behaviors and habits. For example, eating breakfast regularly [5] and consuming meals frequently is associated with a higher diet quality especially if some eating occasions are low in energy [6]. These behavioral aspects of eating are also associated with body mass index (BMI) changes over time [7].

Food choices vary by country and culture. Therefore, locally adapted FFQs are usually based on local nutrition recommendations. Nordic countries have published their own Nutrition Recommendations since 1980 based on current scientific knowledge. The latest Finnish nutritional recommendations (2014) [8] were based on Nordic Nutrition Requirements of 2012 [9], and approved by the National Nutrition Council of Finland. Both guidelines focus on the components of a balanced diet including, for example, a high intake of whole grains and a low intake of sugar-sweetened beverages and also local food groups, such as vegetables, berries, fat-free milk products, and fatty fish. Numerous studies have demonstrated the beneficial impact of Nordic dietary patterns [10,11,12,13]. In particular, Kanerva et al. has shown that the Baltic Sea Diet Score is associated with weight change during 7 years of follow-up, especially in younger women and overweight individuals [13].

Many instruments have been developed to estimate diet quality. In Finland, extensive FFQs based on Nordic and Finnish nutritional recommendations have been developed and have shown reasonable validity [14,15]. Nevertheless, there is increasing evidence that response rates are lower when questionnaires are long [16,17]; thus, a reduction of the number of questions could increase participation rates. Furthermore, some shorter FFQs almost provide similar levels of accuracy as longer FFQs [18,19], but most short FFQs have been developed to estimate the habitual intake of a single nutrient rather than overall diet quality [20,21,22]. Even single item questions on self-rated diet quality may provide a simple method of identifying individuals with the poorest diet quality, but this measure requires further evaluation [23]. Because twin studies are the perfect platform to separate genetic and environmental influences on multiple health behaviors including food or nutrient intake [24,25], short FFQs are essential for inclusion in twin and family studies with a broad health focus.

To our knowledge, only two studies have validated short FFQs for Finnish adults. These are useful tools for estimating nutrient intakes in the primary health care setting [26] and adherence to nutrition recommendations [27]. Inspired by this questionnaire, we constructed a food-based Diet Quality Score (DQS) from a short FFQ to estimate diet quality in a cohort of young adult Finnish twins. The aims of this study were to examine the construct validity of the DQS by examining associations with obesity measures, eating styles and nutrient intakes in twin individuals and by comparing co-twins. 

## 2. Materials and Methods 

### 2.1. Study Population

The main study sample was derived from wave 5 of the population-based FinnTwin16 (FT16) cohort study. The longitudinal FT16 twin cohort study investigates determinants of health-related behaviors and disease in adolescents and young adults [28,29]. In FT16, virtually all twins born in 1975–1979 were identified from the Finnish population register and enrolled in 1991, with baseline assessments of twins and their parents as the twins turned 16 years of age. Follow-up questionnaires were mailed to the twins at ages 17, 18.5 and as young adults, when the participants were on average 24.5 years old. The last follow-up (wave 5) questionnaire was administered as an internet survey during the period 2010–2012, when the participants were in their mid-thirties. The cohort has over 3000 twin pairs, and the invitation was sent to all twins living in Finland regardless of earlier participation. Of the 6132 twins that were contacted, 4414 participated (response rate of 72%).

Subsamples of FT16 have participated in clinical, in-person studies. Such pairs were selected from the whole cohort based on body mass index (BMI) (TwinFat) and physical activity habits (FITFATTWIN). In TwinFat, over 550 monozygotic (MZ) and dizygotic (DZ) twins from BMI-concordant and BMI-discordant pairs completed an intensive metabolic study protocol and assessment of body composition and behavioral traits (questionnaires, interviews, and food diaries) in the period 2002–2013 in Helsinki [30]. The FITFATTWIN study consisted of 23 MZ twin pairs concordant and discordant for physical activity who participated in in-depth clinical examinations and physical activity interviews in Jyväskylä during the period 2011–2012 [31]. TwinFat and FITFATTWIN were studied using partly identical procedures. The FinnTwin16 wave 5 follow-up study plan was approved on 20 April 2010 by the Ethics Committee of the Central Finland Health Care District (Dnro 4/2010). The TwinFat Study plan was approved on 8 August 2008 (Dnro 270/13/03/01/2008) by the Ethics Committee of the Helsinki University Hospital. The FITFATTWIN study plan was approved on 22 March 2011 by the Ethics Committee of the Central Finland Health Care District (Dnro 4U/2011). Written informed consent was obtained from all participants. 

### 2.2. Measurements

#### 2.2.1. Dietary Intake Assessment and Construction of the Food-Based Diet Quality Score (DQS)

##### Diet Quality Score

The short FFQ inquired about the habitual consumption frequency over the previous 12 months for the following 14 food and beverage items: (1) dark bread (rye or crisp bread); (2) mixed flour bread (yeast, oat, or graham bread); (3) white bread (baguette, toast or similar); (4) fruits and berries; (5) vegetables; (6) fish; (7) whole grains (porridge, muesli, dark pasta or rice, whole grain cereals or similar); (8) fast food (hamburgers, pizza, French fries or similar); (9) fat free or reduced-fat milk, sour milk or yoghurt; (10) sugar-sweetened soft drinks or juices; (11) energy drinks; (12) butter; (13) margarine; and (14) vegetable oil. For bread consumption, participants were asked how many slices they usually consume per day. For the other items, food frequency consumption was arranged into five frequency response categories: “not at all”, “few times per month”, “few times per week”, “once a day” and “many times a day”. 

The DQS was constructed to estimate diet quality according to Nordic [9] and Finnish nutrition recommendations, with a focus on fiber, sugar and fat quality [8]. The scoring of the DQS was developed following the approach described by Leppälä et al. [27]. The scoring and cutoff values for individual items are presented in Table 1. Margarine and vegetable oil were combined into one category. No points were given for mixed flour bread. For each of the 12 food categories, one point was given if the specific dietary recommendation was met [8,9]. The points were summed up to calculate an overall score ranging from 0 to 12 points, with a higher score indicating better diet quality and a more favorable diet. For selected analyses, participants were classified to have lower (0–6 points) or higher (7–12 points) diet quality.

##### Food Diaries

TwinFat participants completed a food diary of three consecutive days, while FITFATTWIN twins reported on four days of their dietary intake. The participants were instructed to record in detail all foods and beverages they consumed, using ordinary household measures. They had to fill out these food diaries for one weekend day, and the remaining days were weekdays. The completed food diaries were returned during the clinical examination and were checked for completeness by a researcher or dietitian. 

Nutrient intakes from food diaries were calculated using the Diet 32 software and AivoDiet 2.0.1.2-software (Aivo Ltd., Turku, Finland), which incorporates data from the Fineli^®^ Finnish Food Composition Database (National Institute for Health and Welfare, Nutrition Unit, Helsinki, Finland). Mean nutrient intakes were calculated by taking the mean of the 3 or 4 days. Dietary macronutrient intakes were presented as the percentage of total energy intake (kcal) and micronutrient intakes as grams per 1000 kcal. 

#### 2.2.2. Assessment of Eating Styles

Eating styles were obtained by a questionnaire modified from Keski-Rahkonen et al. [32] that addressed 7 different eating styles as follows: meal frequency, regular eating, health-conscious eating, night eating, external eating, emotional eating and snacking eating styles. Participants were asked to choose one of four options that best characterizes their overall eating style (2 items for assessing meal frequency and 2 items for regular eating styles) and answer 3 items assessing health-conscious eating, 1 item about night eating, 1 item for assessing externally cued eating, 2 items for assessing emotional eating and 5 items for assessing snacking styles, with the response categories “usually”, “often”, “sometimes” or “seldom” (Supplementary Table S1).

#### 2.2.3. Anthropometric Measurements

BMI in kg/m^2^ was calculated from self-reported weight and height. Waist circumference (WC) was self-measured in centimeters midway between the lowest part of the ribs and the upper part of the hip bone. We classified the participants according to the WHO standards for BMI: underweight (BMI < 18.5); normal weight (BMI = 18.5–24.9); overweight (BMI = 25.0–29.9) and obesity (BMI > 30); and for WC: normal (WC < 88 cm for women and <102 cm for men) and abdominal obesity (WC > 88 cm for women and >102 cm for men). The validity of the self-reported BMI, WC, and height measures was assessed previously in a subsample and the intraclass correlations between self-reported and measured measures was high [33].

#### 2.2.4. Assessment of Covariates

Physical activity was assessed by questionnaire, and included 2 items to assess the weekly hours of physical activity: “How often do you exercise in your leisure time?” with 7 response alternatives and “How long do you exercise per occasion?” with 4 possible response alternatives. The intensity of physical activity was assessed by 1 item “Is your physical activity during leisure-time about as strenuous on average as: walking, alternately walking and jogging (slow running), jogging or running”. Physical activity was assessed as leisure time metabolic equivalent units (MET index) and was expressed as the sum score of MET hours per day. The MET index was calculated as the average frequency x duration x intensity [34].

For education, we calculated the years and defined education level as the highest education level achieved. Completed education was categorized as primary and compulsory education (9 years), vocational and academic secondary education (up to 12 years) and tertiary education (>12 years, i.e., university and polytechnic) [35]. 

#### 2.2.5. Derivation of the Total Sample and Subsample

For the present analysis, we included twins with complete data on sex, age, BMI, MET index and education level. We analyzed two samples: (1) the main FT16 sample includes 3592 young adult twin individuals from 1194 complete pairs (764 DZ and 430 MZ pairs); and (2) the subsample with participants from TwinFat and FITFATTWIN includes 249 young adult twins from 105 complete pairs (45 same-sex DZ and, 60 MZ pairs), who returned their food diaries as part of their clinical examination. 

### 2.3. Statistical Analyses

Non-normally distributed variables were log-transformed before the analyses. To describe general characteristics of the main sample and the subsample, continuous variables were presented as means and 95% CIs for age, physical activity, and BMI; and categorical variables as numbers and percentages for age, education, and obesity. Differences between the sample with and without food diaries were determined using the adjusted Wald test for continuous variables and Pearson’s χ^2^ test for categorical variables. 

The association of the DQS with obesity measures, eating styles and nutrient intakes was evaluated in twin individuals, like in any other non-twin population (individual-level analysis) and within twin pairs (within-pair analysis). All individual-level analyses that treated twins as individuals accounted for twin pair clustering by survey methods [36]. We tested that the mean in the DQS for individuals did not differ by zygosity or twin type (same-sex vs. opposite sex) to ensure that they all represent the same base population. For the within-pair analysis, differences between co-twins were calculated by subtracting the value of one twin from that of the other twin, with the twin order in a pair assigned at random. However, for the analysis on twin pairs discordant for diet quality, twin pairs were sorted according to lower and higher diet quality. 

The association between the DQS and obesity measures were presented as multiple regression models adjusted for age, sex, education and physical activity. Regression coefficients and 95% CIs are shown for individual-level analysis and within-twin pair analysis, for all twin pairs and by zygosity. The association between the DQS and overweight, obesity and abdominal obesity were presented as odds ratios (OR) and 95% CIs. Conditional regression analyses were calculated for the within-twin pair analysis. ORs were adjusted by age, sex, education and physical activity and were calculated for individuals and within-twin pairs, for all twin pairs and by zygosity. 

The association between the DQS and eating styles were calculated using multiple regression models adjusted for age, sex, BMI, education and physical activity levels with the DQS as the dependent variable and the eating style as the independent variable. For the individual-level analysis, adjusted means and their 95% CIs are shown for the 4 categories of eating styles. For the within-pair analysis, regression coefficients and 95% CIs are shown for all twin pairs and by zygosity. 

Nutrient intakes between lower (1–6 points) and higher (7–12 points) adherence groups were evaluated by the adjusted Wald test. Nutrient intakes between twin pairs discordant for diet quality (defined as at least 3 points difference in the DQS between the twins in the pair) were evaluated by the paired t-test (for normally distributed variables) or by the Wilcoxon signed-rank test (for non-normally distributed variables). Correlations between the DQS and nutrient intakes were evaluated by Pearson’s partial correlations adjusted for age, sex, BMI, physical activity and education level in individual-level and within-pair analysis (all pairs and by zygosity). All statistical analyses were carried out by using Stata 14.1 statistical package (StataCorp, College Station, TX, USA) (www.stata.com). A value of *p* < 0.05 was considered significant.

## 3. Results

### 3.1. General Characteristics of the Main Study Sample

In the main sample, approximately 39% individuals had a lower DQS and 61% had a higher DQS (Table 2). Participants with a higher DQS were more physically active, were more highly educated, had lower BMIs and WCs and were less often overweight, obese and abdominal obese. Women had a higher mean DQS than men (7.5 for women and 6.5 for men, *p* < 0.001).

The subsample with food diaries included more men and overweight individuals and was more educated when compared to the sample without food diaries, but there were no differences with respect to age, physical activity and diet quality. 

### 3.2. Obesity and the DQS in Twin Individuals and within Twin Pairs

A higher DQS was inversely associated with BMI (*β* = −0.12, per one-unit increase in DQS, *p* < 0.001), WC (*β* = −0.34, *p* < 0.001) and a lower odds of being overweight and abdominally obese (OR = 0.95, per one-unit increase in DQS, *p* = 0.004 for overweight, OR = 0.88, *p* < 0.001) (Table 3). 

When analyzing males and females separately, the DQS was inversely associated with BMI in females (*β* = −0.17, *p* = 0.003) and with WC in males (*β* = −0.42, *p* = 0.003). The DQS was associated with a lower odds of being overweight (OR = 0.94, *p* = 0.03 for men and OR = 95, *p* = 0.04 for women) and abdominal obese (OR = 91, *p* = 0.02 for men and OR = 0.93, *p* < 0.05 for women) in both men and women.

The DQS was not associated with obesity measures or the odds of overweight/obesity or abdominal obesity when comparing twin pairs, and neither when stratifying the within-pair analyses by sex.

### 3.3. Eating Styles and the DQS in Twin Individuals

Higher health-conscious eating and higher meal frequency were associated with a higher mean DQS, while snacking frequently, engaging in external eating and consoling oneself more often by eating and drinking were associated with a lower mean DQS (Table 4). Restrictive or overeating styles as well as waking up at night to eat were not associated with the DQS. When stratifying the analyses by sex, there were fewer associations in men than in women. Specifically, associations with external eating, consoling oneself more often by eating and drinking, and replacing meals for snack were significant in women but not in men, except rewarding oneself with good food, which was significant in men but not in women. The effect sizes were as follows: external eating (*β* = 0.05, *p* = 0.7 for men and *β* = 0.44, *p* = 0.003 for women), console oneself more often by eating and drinking (*β* = −0.03, *p* = 0.9 for men and *β* = 0.30, *p* = 0.005 for women), replacing meals for snacks (*β* = 0.35, *p* = 0.07 for men and *β* = 0.62, *p* < 0.001 for women), and rewarding oneself with good food (*β* = −0.20, *p* < 0.05 for men and *β* = 0.15, *p* = 0.07 for women). 

### 3.4. Eating Styles and the DQS within Twin Pairs

Within all twin pairs (both MZ and DZ) pooled together, health-conscious eating styles, breakfast regularity, frequency of meals per day and regular eating styles were associated with a higher DQS (Table 5). More frequent snacking was associated with lower diet quality. Not being externally influenced to eat was associated with a higher DQS when comparing twin pairs. 

Within DZ twin pairs, we observed the same associations between eating styles and the DQS as in the twin pair analysis pooling all the twins that was described above, with an exception in external eating, which was not associated with the DQS in DZ twin pairs. 

When analyzing male same-sex, female same-sex and opposite-sex pairs separately, we observed that external eating was associated with a higher DQS in all same-sex DZ pairs (*β* = 0.41, *p* = 0.03) and the effect sizes were about equal in magnitude when analyzing female DZ pairs (*β* = 0.41, *p* = 0.1) and male DZ pairs (*β* = 0.45, *p* = 0.13) separately, but not in opposite-sex twin pairs (*β* = 0.02, *p* = 0.9). In addition, snacking between meals was associated with a lower DQS in all same-sex DZ twin pairs (*β* = −0.39, *p* = 0.001) and male DZ pairs (*β* = −0.56, *p* = 0.001), but not in female DZ pairs (*β* = −0.24, *p* = 0.2) or opposite-sex twin pairs (*β* = 0.16, *p* = 0.3). Watching TV while eating was associated with a higher DQS in all same-sex DZ pairs (*β* = 0.49, *p* < 0.001) and male DZ pairs (*β* = 0.84, *p* < 0.001) but not in female DZ pairs (*β* = 0.21, *p* = 0.2) or opposite-sex DZ twin pairs (*β* = 0.08, *p* = 0.5). Some more associations were not significant in either male pairs or female pairs: frequency of meals per day (*β* = 0.36, *p* = 0.2 for male pairs and *β* = 1.29, *p* < 0.001 for female pairs), replacing meals for snacks (*β* = 0.25, *p* = 0.3 for male pairs and *β* = 0.57, *p* = 0.004 for female pairs), and higher consumption in the evening (*β* = 0.31, *p* = 0.13 for male pairs and *β* = 0.49, *p* = 0.005 for female pairs). 

When the analysis was limited to MZ twin pairs, results were similar to all twin pair analysis; however, snacking between meals and external eating styles were not associated with the DQS within MZ twin pairs. Consoling oneself by eating and drinking more often was associated with the DQS within MZ twin pairs. 

### 3.5. Nutrient Intake and the DQS in Twin Individuals

Nutrient intake differed between individuals with lower and higher diet quality (Table 6). Mean nutrient intakes particularly differed significantly for total fat, saturated fat, sucrose, fiber and magnesium. There were no differences in the means of total energy intake, carbohydrates, protein and iron between individuals with lower and higher diet quality. 

Magnesium, fiber and protein intake were positively correlated with the DQS (r_max_ = 0.30 for magnesium, *p* < 0.001). Total fat, saturated fat and sucrose intake were inversely correlated with the DQS (r_max_ = −0.24 for saturated fat and sucrose, *p* < 0.001).

### 3.6. Nutrient Intake and the DQS within Twin Pairs

Twin pairs discordant for the DQS differed in the mean intake of saturated fat, which was lower in the twin with a higher DQS (Table 7). Magnesium and fiber intakes were positively correlated with the DQS within all twin pairs (both MZ and DZ) pooled together in the analysis (r = 0.28 for magnesium, *p* = 0.005). Within-pair analyses limited to DZ twin pairs revealed results largely consistent with those obtained from within-pair analyses of all twin pairs, with positive correlations for magnesium and fiber intakes (r = 0.40 for magnesium, *p* = 0.01); furthermore, saturated fat was inversely correlated with the DQS (r = −0.36, *p* = 0.02). In contrast, in within-pair analyses of MZ twin pairs, sucrose intake was inversely correlated with the DQS (r = −0.42 for sucrose, *p* = 0.002). 

## 4. Discussion

This study describes the development and validation of a food-based Diet Quality Score (DQS), derived from a short FFQ that was specifically developed for the FinnTwin16 survey. Our main findings demonstrated that a higher DQS was associated with lower BMI and WC and with a lower risk of being overweight and abdominal obese during young adulthood. A higher DQS was also associated with healthier eating styles and lower intakes of sucrose. When we examined the relationship within twin pairs, the twin with the higher DQS had healthier eating styles and healthier nutrient intakes. 

Our results support the associations between diet quality and obesity measures. Previous studies have also shown an association between different scores that reflect diet quality and the risk of overweight/obesity [4,37]. As a novel aspect, in the present study, a diet quality score derived from a short FFQ that only asked about the usual consumption frequency of 14 foods and beverages showed associations with obesity measures in a similar magnitude as those diet quality scores derived from more extensive FFQs [38]. Notably, the associations between diet quality and obesity measures that were observed in the general population were not observed when comparing twin pairs, suggesting that the association might not be causal but rather due to confounding by socioeconomic or genetic factors. 

The DQS was also associated with several eating styles from our questionnaire (Supplementary Table S1). Healthier eating styles (health-conscious eating, less emotional eating and less external eating) were positively associated with the DQS. Our results are novel because very few studies to date have addressed the associations between various eating styles and diet quality simultaneously. In line with previous observations, emotional and external eating styles were associated with low diet quality [39]. In previous research, emotional and external eating were associated with higher intakes of snacks and fast food, while restrained eating was associated with higher intake of sweet foods. External eating mediated the association between depression and higher intake of snack and fast food. This previous study has further shown that eating styles and depression are independently associated with a poorer diet quality [40].

Previous research has largely focused on eating frequency (meal and snack frequency) [41]. We found that a higher DQS was associated with an increased eating frequency. Our results were consistent with recent research, in which participants with an increased meal frequency and decreased snacking styles had higher diet quality [42,43]. The impact of snacking styles on diet quality remains unclear, and is complicated by the fact that there is no universally accepted definition of snacking [44]. Some studies have found that increased snack frequency was associated with better diet quality [45,46]. The evidence for breakfast is less clear, with some studies showing that eating breakfast regularly is associated with better diet quality [47] and other studies showing the opposite [48]. In future research, the quality of snack choices needs to be considered more carefully [49].

The newly developed DQS shows associations with obesity measures, eating styles and key nutrients in the expected direction. Participants with a higher DQS showed a more beneficial nutrient intake in key nutrients such as fat, saturated fat, protein, sucrose, fiber and magnesium. Correlations of the DQS with energy intake, carbohydrates and some micronutrients were weak or nonexistent. Nutrient associations followed a similar trend of other validation studies conducted elsewhere [26,27,50,51,52], where most of the beneficial nutrient intakes were correlated with diet quality. Our weaker associations with some nutrients could have arisen from the fact that we examined a large number of nutrients in relation to a diet quality score derived from a short FFQ consisting of just a few food items. Previously validated other short FFQs have mainly focused on a single nutrient [20,21,22]. In the present study, a higher diet quality as indicated by a higher DQS was most closely associated with lower intakes of saturated fat, lower intakes of sucrose and higher intakes of magnesium.

There are some important limitations to this study. First, there are limitations associated with the use of a short FFQ to estimate diet quality. Generally, short screeners for diet quality only assess the frequency of consumption but do not capture portion sizes. Correlation coefficients against other dietary assessment methods are lower when the number of items on a FFQ is lower [53]. Therefore, such brief instruments are less accurate than longer FFQs. However, short dietary assessment methods are useful in large studies that focus on a variety of exposure and that need to keep the participant burden minimal. The DQS in the current study had a focus on fiber, sugar and fat, which was also reflected in the correlations with nutrient intakes. Furthermore, as with all studies using self-reported dietary intake data, study participants, in particular females and individuals with obesity, tend to give socially desirable answers to questions on diet, either by underreporting behaviors considered unhealthy or by overreporting normative ones [54,55,56]. This may introduce systematic errors, which can confound associations between dietary exposures and health outcomes [57]. Second, only participants from the subsample (TwinFat and FITFATTWIN) completed the food diaries; therefore, we have less power for the nutrient analysis than for the analysis with eating styles due to a reduced sample size. A limited sample size also means insufficient statistical power for addressing genetic confounding by limiting the analyses to MZ twin pairs. Third, there was a time lag of approximately 4 years between the food diaries and the date when the short FFQ was filled in, and during these years participant’s diet could have changed over time. However, previous research has shown that dietary patterns are reasonably stable over time [58] and that dietary patterns identified in the recent past provide useful information about current dietary patterns [59,60]. Finally, we compared the food-derived DQS with nutrient intake data from food diaries. Due to the different levels of dietary assessment (food-based vs. nutrient-based), we would not expect high correlations with all nutrients even in the situation with near perfect validity of the score, even though the correlations were in the expected direction. 

Our study has some important strengths. The developed diet quality score derived from a short FFQ is an easy-to-use tool for study participants and can be used to differentiate between individuals and twin pairs with higher and lower diet quality. The DQS could be used to screen diet quality in other Westernized countries with similar dietary recommendations as the Nordic recommendations. Similar tools have been developed in other populations, such as the 14 point Mediterranean Diet Adherence Screener [50] and a 14 item FFQ to evaluate dietary patterns in relation with coronary risk in a French population [61]. Many existing short screeners have focused on the assessment of a single nutrient [20,21,22] but we aimed at developing a tool that reflects a more comprehensive picture of the diet, with a focus on fiber, sugar and fat quality. The present study’s strength further includes the simultaneous assessment of eating styles that allowed the assessment of an additional aspect of dietary behavior. An additional strength was the rather large sample of twin pairs. Twin studies have been useful to estimate the heritability of traits and to control for genetic and familial effects; however, twins can also be analyzed at the individual level, like any other non-twin sample.

## 5. Conclusions

The derived DQS showed associations with obesity measures, eating styles and nutrient intakes in the expected direction, showing the construct validity of the constructed DQS [62]. A higher DQS was associated with a lower BMI and a lower WC and additionally with a lower risk of overweight and abdominal obesity. The DQS reflected eating styles and intakes of key nutrients related to diet quality. A higher diet quality was associated with increased eating frequency, breakfast eating, and health-conscious eating, less emotional eating and less external eating styles and with healthier nutrient intakes, particularly a higher magnesium intake and a lower saturated fat and sucrose intake. Therefore, the DQS derived from a short FFQ represents a quick instrument for screening Finnish and similar Western populations for diet quality. Future studies should examine the interrelationships between the DQS with other variables, such as social desirability and nutrient biomarkers. Future studies should further explore why behaviors related to food intake are related to diet quality and research on meal patterns and health outcomes need to consider diet quality as a potential confounder.

## Figures and Tables

**Table 1:** Overview of the development of the food-based diet quality score.

Food Category	Recommendation [8,9]	Frequency	Score
Dark bread (rye or crisp bread)	Carbohydrate intake should be between 45–60 E%. Intake of dietary fiber should be at least 25–35 g/d or 3 g/MJ. Exchange white bread with whole grain alternatives	2 or more slices per day	1p

		0 or 1 slice per day	0p

White bread, baguette, toast or something similar		0 slices per day	1p
		2 or more slices per day	0p
Fruits and berries	Eat vegetables, fruits and berries frequently (a minimum of 500 g/day, excluding potatoes). Important source of dietary fiber intake.	Use of fruits and berries daily or several times per day	1p
		Use of fruits and berries less than daily	0p
Vegetables		Use of vegetables daily or several times per day	1p
		Use of vegetables less than daily	0p
Fish	Eat fish (of different kinds) two to three times a week. N-3 fatty acids should provide at least 1 E%.	Use of fish at least a few times per week	1p
		Use of fish never or only a few times per months or more rarely	0p
Whole grains (porridge, muesli, dark pasta or rice, whole grain cereals or something similar)	Eat wholegrain cereals (bread, porridge, pasta, etc.) several times a day. Prefer fiber-rich and low-salt products	Use of whole-grain foods daily or several times per day	1p
		Use of whole-grain foods less than daily	0p
Fast food (hamburgers, pizza, French fries or something similar)	Saturated fatty acids < 10 E%. Avoid products made of refined flour with plenty of hard fat and sugar. Exchange. frying food to boiling and cooking in the oven	Use of fast food never or a few times per months or more rarely	1p
		Use of fast food a few times per week or more often	0p
Fat free or reduced-fat milk, sour milk or yoghurt	Consume fat-free/low-fat milk products daily (5–6 dL/day). Protein 10–20 E%	Use of fat free or reduced fat milk products at least daily	1p
		Use of fat free or reduced fat milk products less than daily	0p
Sugar-sweetened soft drinks or juices	Intake of added sugars should be kept < 10 E%. Consumption of sugar-sweetened beverages should be limited	Use of sugar-sweetened soft drinks or juices never or seldom (few times per months)	1p
		Use of sugar-sweetened soft drinks a few times per week or more often	0p
Energy drinks	Intake of added sugars should be kept < 10 E%. Consumption of sugar-sweetened beverages should be limited	Use of energy drinks never or seldom (few times per months)	1p
		Use of energy drinks a few times per week or more often	0p
Butter	Saturated fatty acids < 10 E%. Exchange butter with vegetable oil-based fat spreads and oils	Use of butter never or seldom (few times per months)	1p
		Use of butter a few times per week or more often	0p
Margarine	Monounsaturated fatty acids 10–20 E%. Polyunsaturated fatty acids 5–10 E%	Use of margarine or vegetable oil daily or several times per day	1p
Vegetable oil		Use of margarine or vegetable oils less than daily	0p

**Table 2:** General characteristics of the study sample according to the food-based diet quality score.

	Lower Diet Quality (Score: 0–6)	High Diet Quality (Score: 7–12)	*p*-Value
Sample size, *n*	1395	2197	
Monozygotic twin individuals *	420 (32)	696 (33)	0.1
Dizygotic twin individuals *	908 (68)	1414 (67)	
Age in years	34.1 (34.0, 34.1)	34.2 (34.1, 34.2)	0.03
Sex			<0.001
Female	616 (44)	1398 (64)	
Men	779 (56)	799 (36)	
Education level			<0.001
Compulsory education	88 (6)	43 (2)	
Vocational and academic	727 (52)	856 (39)	
secondary education			
Tertiary education	580 (42)	1298 (59)	
Physical activity			<0.001
Metabolic equivalent			
(MET) in hours per day	2.8 (2.6, 3.0)	4.2 (4.0, 4.4)	
BMI in kg/m^2^	25.4 (25.2, 25.7)	24.2 (24.0, 24.4)	<0.001
Body weight category			
Underweight	21 (2)	34 (1.5)	
Normal weight	715 (51)	1404 (64)	
Overweight	477 (34)	590 (27)	
Obesity	182 (13)	169 (7.5)	
Waist circumference (WC) in cm **	88.7 (87.9, 89.4)	84.0 (83.5, 84.6)	<0.001
Abdominal obesity category **			
Normal	1053 (77)	1789 (83)	
Abdominal obesity	313 (23)	365 (17)	

Data are presented as numbers (%) or means (and confidence intervals). *p*-values were determined by the adjusted Wald test for continuous variables and Pearson’s χ^2^ test for categorical variables and corrected for clustering of twin pairs by survey methods. The mean diet quality score in the lower diet quality group was 4.9 (95% CI: 4.8, 4.9) and in the higher diet quality group was 8.4 (95% CI: 8.4, 8.5). * Sample size is smaller than the total sample due to twins of unknown zygosity. ** Sample size smaller due to missing values.

**Table 3:** Associations between the food-based diet quality score (DQS) and obesity measures and risk of overweight, obesity and abdominal obesity.

	*n*	Beta Coefficients (95% CI)		Odds Ratio (95% CI)		
		Body Mass Index	Waist Circumference	Overweight	Abdominal Obesity	Obesity
Individual-level analyses	*n* (individuals)					
All individuals	3592	0.12 (−0.20, −0.05) *	−0.34 (−0.55, −0.14) *	0.95 (0.87, 0.93) **	0.88 (0.84, 0.91) *	0.95 (0.90, 1.01)
Within-twin pair analyses	*n* (pairs)					
All twin pairs	1237	0.00 (−0.09, 0.09)	−0.04 (−0.32, 0.24)	1.02 (0.94, 1.11)	1.01 (0.92, 1.12)	1.05 (0.92, 1.19)
Dizygotic twin pairs	764	0.02 (−0.11, 0.15)	0.04 (−0.33, 0.42)	1.01 (0.91, 1.11)	1.03 (0.92, 1.15)	1.08 (0.93, 1.25)
Monozygotic twin pairs	430	−0.03 (−0.15, 0.09)	−0.17 (−0.60, 0.27)	1.03 (0.92, 1.15)	0.95 (0.75, 1.19)	1.04 (0.75, 1.44)

Data are beta-coefficients for a one-unit increase in DQS, from multivariate models with respective 95% confidence intervals. Odds ratios for a one-unit increase in DQS are presented with respective 95% confidence intervals. All models are adjusted by sex, age, physical activity (expressed as MET hours per day) and education level. * *p* < 0.001. ** *p* < 0.01. The sample size is smaller due to some missing values for waist circumference.

**Table 4:** Adjusted mean food-based diet quality score (DQS) by categories of eating styles in the total sample (*n* = 3592).

Eating Style	Variable	Categories				*p*-Value
		Every Morning	5 to 6 Times a Week	2 to 4 Times a Week	Once a Week or Less Frequent	
Meal Frequency	How often do you eat breakfast?	7.5 (7.4, 7.5)	6.8 (6.6, 7.0)	6.2 (6.0, 6.4)	5.9 (5.7, 6.1)	<0.001
		1 to 2 times	3 to 4 times	5 to 6 times	7 times or more often	
	How often in a day do you usually eat?	5.8 (5.6, 6.1)	6.8 (6.7, 6.8)	7.7 (7.6, 7.8)	7.8 (7.1, 8.4)	<0.001
Regular eating		I eat very regularly	I eat quite regularly	I eat quite irregularly	I eat very irregularly	
	Regularity of your eating habits	7.8 (7.6, 7.9)	7.2 (7.1, 7.2)	6.1 (5.9, 6.3)	5.3 (5.0, 5.6)	<0.001
		It is easy for me to eat pretty as much I need	Quite often I eat more than needed	I often try to restrict my eating	Sometimes I’m on a strict diet, at other I overeat	
	Restrictive/overeating style	7.2 (7.1, 7.3)	6.8 (6.6, 6.9)	6.9 (6.6, 7.1)	6.9 (6.6, 7.2)	0.1
Health-conscious eating style		Usually	Often	Sometimes	Seldom	
	I tend to eat healthily	7.7 (7.6, 7.8)	6.9 (6.8, 7.0)	5.5 (5.4, 5.7)	5.2 (4.7, 5.6)	<0.001 <0.001 <0.001
	I avoid greasy meals	7.9 (7.8, 8.1)	7.5 (7.4, 7.6)	6.5 (6.5, 6.8)	5.9 (5.7, 6.1)	
	I avoid calories	7.8 (7.6, 8.0)	7.6 (7.5, 7.7)	7.0 (6.9, 7.1)	6.5 (6.4, 6.6)	
Night eating style	At nights I wake up to eat	7.6 (6.8, 8.5)	5.5 (4.7, 6.4)	6.7 (6.3, 7.1)	7.0 (7.0, 7.1)	0.7
External eating style	I eat tempted to the advertisements	6.2 (5.5, 6.9)	6.2 (5.7, 6.6)	6.8 (6.6, 6.9)	7.1 (7.1, 7.2)	0.005
Emotional eating style	I reward myself with good food	6.9 (6.5, 7.2)	6.8 (6.6, 7.0)	7.1 (7.0, 7.2)	7.0 (6.9, 7.1)	0.2
	I console myself by eating or drinking	6.4 (5.9, 6.9)	6.8 (6.5, 7.1)	7.0 (6.9, 7.1)	7.1 (7.0, 7.2)	0.005
Snacking eating style	During meal times I eat sufficiently—I don’t need to snack between meals	7.2 (7.1, 7.3)	7.1 (7.0, 7.2)	6.4 (6.3, 6.6)	6.4 (6.1, 6.8)	<0.001 <0.001 <0.001 <0.001 <0.001
	I replace my meals with snacks	5.9 (5.4, 6.5)	6.1 (5.8, 6.3)	6.9 (6.8, 7.0)	7.3 (7.2, 7.4)	
	I eat most in the evenings	6.1 (5.9, 6.3)	6.5 (6.4, 6.7)	7.0 (6.9, 7.1)	7.4 (7.3, 7.5)	
	My food consumption is highest in the evening	6.2 (5.9, 6.5)	6.6 (6.4, 6.7)	7.0 (6.9, 7.1)	7.3 (7.2, 7.4)	
	While I am eating, I watch TV	6.6 (6.4, 6.9)	6.5 (6.4, 6.7)	7.2 (7.1, 7.3)	7.3 (7.2, 7.4)	

Data are adjusted means from multivariable models with respective confidence intervals. All models are adjusted for sex, age, body mass index, physical activity (expressed as MET hours per day) and education level.

**Table 5:** Association between the food-based diet quality score (DQS) and eating styles within twin pairs.

Eating Style	Variable	All Twin Pairs (n = 1194)	*p*-Value	Dizygotic Twin Pairs (n = 764)	*p*-Value	Monozygotic Twin Pairs (n = 430)	*p*-Value
Meal Frequency	How often do you eat breakfast?	−0.46 (−0.56, −0.36)	<0.001	−0.46 (−0.59, −0.34)	<0.001	−0.53 (−0.70, −0.41)	<0.001
	How often in a day do you usually eat?	0.76 (0.57, 0.94)	<0.001	0.81 (0.57, 1.05)	<0.001	0.80 (0.45, 1.15)	<0.001
Regular eating	Regularity of your eating habits Restrictive/overeating style	−0.72 (−0.86, −0.58) −0.16 (−0.30, −0.02)	<0.001 0.02	−0.80 (−0.98, −0.62) −0.20 (−0.37, −0.02)	<0.001 0.03	−0.91 (−1.15, −0.66) −0.16 (−0.41, 0.09)	<0.001 0.2
Health-conscious eating style	I tend to eat healthily	−0.82 (−0.97, −0.68)	<0.001	−0.92 (−1.09, −0.74)	<0.001	−0.59 (−0.83, −0.34)	<0.001
	I avoid greasy meals	−0.58 (−0.70, −0.47)	<0.001	−0.73 (−0.88, −0.59)	<0.001	−0.29 (−0.48, −0.11)	0.002
	I avoid calories	−0.43 (−0.55, −0.30)	<0.001	−0.52 (−0.67, −0.36)	<0.001	−0.22 (−0.42, 0.02)	0.03
Night eating style	At nights I wake up to eat	−0.07 (−0.44, 0.30)	0.7	−0.02 (−0.51, 0.47)	0.9	−0.13 (−0.68, 0.43)	0.7
External eating style	I eat tempted to the advertisements	0.20 (0.01, 0.39)	0.04	0.21 (−0.05, 0.46)	0.1	0.20 (−0.08, 0.48)	0.2
Emotional eating style	I reward myself with good food	0.10 (−0.04, 0.24)	0.2	0.07 (−0.11, 0.25)	0.5	0.17 (−0.05, 0.40)	0.1
	I console myself by eating or drinking	0.16 (0.00, 0.32)	0.05	0.11 (−0.10, 0.32)	0.3	0.25 (0.01, 0.50)	0.04
Snacking eating style	During meal times I eat sufficiently—I don’t need to snack between meals	−0.25 (−0.38, −0.12)	<0.001	−0.34 (−0.50, −0.18)	<0.001	−0.01 (−0.23, 0.20)	0.9
	I replace my meals with snacks	0.41 (0.25, 0.57)	<0.001	0.45 (0.24, 0.66)	<0.001	0.32 (0.07, 0.58)	0.01
	I eat most in the evenings	0.41 (0.30, 0.53)	<0.001	0.48 (0.33, 0.63)	<0.001	0.28 (0.09, 0.46)	0.003
	My food consumption is highest in the evening	0.40 (0.27, 0.54)	<0.001	0.40 (0.22, 0.57)	<0.001	0.42 (0.20, 0.64)	<0.001
	While I am eating, I watch TV	0.25 (0.12, 0.37)	<0.001	0.27 (0.11, 0.43)	0.001	0.23 (0.02, 0.43)	0.03

Data are beta-coefficients for one-category differences within twin pairs from multivariable models with respective 95% confidence intervals. All models are adjusted by sex, and within-pair differences in the following variables: age, body mass index, physical activity physical activity (expressed as MET hours per day) and education level.

**Table 6:** Association between the food-based diet quality score (DQS) and nutrient intakes in twin individuals from the subsample with food diaries.

	Lower DQS (score: 0–6) (*n* = 93)	Higher DQS (Score: 7–12) (*n* = 156)		Correlation Coefficient (*n* = 249)	
	**Means (95% CIs)**	**Means (95% CIs)**	***p*-Value**	**r**	***p*-Value**
Total energy intake (kcal)	2246 (2113, 2379)	2149 (2044, 2255)	0.3	0.00	>0.9
Carbohydrates (%)	46.3 (44.9, 47.6)	46.2 (44.8, 47.6)	0.9	–0.01	0.9
Protein (%)	16.5 (15.7, 17.4)	17.6 (16.8, 18.4)	0.06	0.14	0.02
Total fat (%)	33.8 (32.7, 34.9)	31.7 (30.5, 32.9)	0.01	–0.18	0.003
Saturated fat (%)	13.0 (12.5, 13.4)	12.0 (11.4, 12.6)	0.01	–0.24	<0.001
Sucrose (%)	11.0 (9.9, 12.1)	9.2 (8.4, 10.0)	0.005	–0.24	<0.001
Fiber (per 1000 kcal)	7.9 (7.3, 8.5)	9.9 (9.1, 10.7)	<0.001	0.23	0.001
Cholesterol (per 1000 kcal)	141.1 (127.3, 154.9)	124.1 (112.9, 135.3)	0.02	–0.15	0.06
Folate (per 1000 kcal)	126.8 (115.3, 138.3)	140.5 (130.8, 150.2)	0.01	0.11	0.1
Calcium (per 1000 kcal)	496.9 (457.4, 536.5)	584.4 (549.9, 619.0)	0.001	0.14	0.05
Iron (per 1000 kcal)	5.9 (5.4, 6.4)	6.1 (5.8, 6.4)	0.2	0.07	0.3
Vitamin C (per 1000 kcal)	46.2 (39.5, 52.9)	56.3 (50.2, 62.4)	0.01	0.12	0.1
Magnesium (per 1000 kcal)	161.8 (153.5, 170.2)	187.0 (179.9, 194.1)	<0.001	0.30	<0.001

Adjusted Wald test and Pearson’s partial correlations adjusted for sex, age, body mass index, physical activity physical activity (expressed as MET hours per day) and education level. *p*-values were *corrected* for *clustering* of *twin pairs* by survey methods.

**Table 7:** Associations between the food-based diet quality score (DQS) and nutrient intakes within twin pairs in the subsample with food diaries.

	Twins Discordant for Diet Quality ^1,2^			Correlations within Twin Pairs ^3^					
	Twin with the Lower DQS (*n* = 31)	Twin with the Higher DQS (*n* = 31)		All Twin Pairs (*n* = 105)		Same-Sex Dizygotic Twin Pairs (*n* = 45)		Monozygotic Twin Pairs (*n* = 60)	
	Means (95% CIs)	Means (95% CIs)	*p*-Value	r	*p*-Value	r	*p*-Value	r	*p*-Value
Total energy intake (kcal)	2288 (2014, 2562)	2161 (1884, 2437)	0.4	−0.11	0.3	0.11	0.5	−0.25	0.06
Carbohydrates (%)	45.1 (42.2, 48.0)	46.5 (43.7, 49.3)	0.24	0.04	0.7	0.19	0.3	−0.18	0.2
Protein (%)	16.6 (15.0, 18.3)	17.0 (15.3, 18.8)	0.84	0.12	0.2	0.13	0.4	0.23	0.1
Total fat (%)	33.4 (31.5, 35.3)	31.2 (28.8, 33.6)	0.09	−0.18	0.08	−0.30	0.06	−0.06	0.7
Saturated fat (%)	13.0 (12.1, 13.8)	11.4 (10.3, 12.4)	0.009	−0.24	0.05	−0.36	0.02	−0.12	0.4
Sucrose (%)	10.1 (8.2, 11.9)	9.1 (7.3, 10.9)	0.3	−0.18	0.07	0.05	0.8	−0.42	0.002
Fiber (per 1000 kcal)	7.8 (6.6, 9.1)	9.0 (7.8, 10.2)	0.08	0.22	0.03	0.37	0.02	0.06	0.7
Cholesterol (per 1000 kcal)	120.0 (104.5, 135.5)	107.2 (94.9, 119.5)	0.1	0.00	>0.9	0.03	0.8	−0.07	0.7
Folate (per 1000 kcal)	136.1 (108.5, 163.6)	126.7 (111.7, 141.7)	0.6	0.09	0.4	0.14	0.4	0.08	0.5
Calcium (per 1000 kcal)	492.2 (417.5, 567.0)	519.4 (434.5, 604.3)	>0.9	0.14	0.2	0.20	0.2	0.05	0.7
Iron (per 1000 kcal)	6.0 (4.8, 7.2)	5.6 (5.1, 6.1)	0.7	−0.03	0.8	−0.05	0.8	−0.02	0.8
Vitamin C (per 1000 kcal)	49.9 (37.1, 62.7)	49.5 (38.3, 60.7)	0.8	0.17	0.09	0.28	0.08	0.07	0.6
Magnesium (per 1000 kcal)	160.8 (147.0, 174.5)	175.3 (158.7, 191.9)	0.2	0.28	0.005	0.40	0.01	0.15	0.3

^1^ Paired t-test (normally distributed variables) or Wilcoxon signed rank test (non-normally distributed variables). ^2^ Twins discordant for the diet quality score. Discordance is defined as a within-pair difference in the diet quality score of ≥ 3 points. ^3^ Pearson’s partial correlations adjusted for sex, and within-pair differences in the following variables: age, body mass index, physical activity physical activity (expressed as MET hours per day) and educational level.

## Supplementary Materials

The following are available online at https://www.mdpi.com/2072-6643/11/11/2561/s1, Supplementary Table S1. Questions and answer categories for eating styles
