# Supplementary material for: Development of a Food-Based Diet Quality Score from a Short FFQ and Associations with Obesity Measures, Eating Styles and Nutrient Intakes in Finnish Twins
Source: Nutrients. 2019 Oct 23;11(11):2561. doi: 10.3390/nu11112561 (PMC6893528; doi:10.3390/nu11112561)
Supplement: Supplementary file 1 [file nutrients-11-02561-s001.pdf]

Supplementary Table S1. Questions and answer categories for eating styles

EATING STYLE	QUESTIONS
**Meal frequency**	How often do you eat breakfast? (Sandwiches, porridge, yoghurt or something similar)<br>- Every morning<br>- 5 to 6 times a week<br>- 2 to 4 times a week<br>- Once a week or less frequent<br>How often in a day do you usually eat? (Take also the snacks into account)<br>- 1 to 2 times<br>- 3 to 4 times<br>- 5 to 6 times<br>- 7 times or more often
**Regular eating**	Which of the alternatives mentioned below describes best the regularity of your eating habits? (Regularity of your eating habits)<br>- I eat very regularly<br>- I eat quite regularly<br>- I eat quite irregularly<br>- I eat very irregularly<br>Which of the alternatives mentioned below describes you best? (Restrictive/overeating style)<br>- It is easy for me to eat pretty as much I need<br>- Quite often I eat more than needed<br>- I often try to restrict my eating<br>- Sometimes I'm on a strict diet, at other I overeat
**Health-conscious eating style**	I tend to eat healthily (Usually to Seldom)<br>I avoid greasy meals (Usually to Seldom)<br>I avoid calories (Usually to Seldom)
**Night eating style**	At nights I wake up to eat (Usually to Seldom)
**External eating style**	I eat tempted to the advertisements (Usually to Seldom)
**Emotional eating style**	I reward myself with good food (Usually to Seldom)<br>I console myself by eating or drinking (Usually to Seldom)
**Snacking eating style**	During meal times I eat sufficiently – I don't need to snack between meals (Usually to Seldom)<br>I replace my meals with snacks (Usually to Seldom)<br>I eat most in the evenings (Usually to Seldom)<br>My food consumption is highest in the evening (Usually to Seldom)<br>While I am eating, I watch TV (Usually to Seldom)
